# Heads up for concussion, what do emergency physicians know? A scoping review

**DOI:** 10.1186/s13102-025-01091-9

**Published:** 2025-03-27

**Authors:** Adam Gowdy, Neil Heron

**Affiliations:** 1 School of Medicine, Dentistry and Biomedical Sciences, Queen’s University Belfast, Belfast, BT12 6BA UK; 2 Centre for Public Health, Queen’s University Belfast, Belfast, BT12 6BA UK; 3 School of Medicine, NIHR, Keele University, Newcastle, ST5 5BG UK

**Keywords:** Concussion, Mild traumatic brain injury, Head injury, Medical education, Emergency medicine, Sports related concussion, Concussion knowledge

## Abstract

**Introduction:**

Concussion is a common condition, with sources estimating between 1.2% and 6.6% of all ED presentations are related to head injury, and concussion has significant healthcare costs. In April 2023, the UK Government published guidelines for diagnosis and management of concussions in grassroots sport, recommending anyone that has sustained a suspected concussion has a same day review by an appropriate healthcare professional. It is therefore essential that emergency medicine physicians have the required knowledge and use current clinical practice guidelines in managing suspected concussions presenting to their departments. This scoping review aims to review the current literature regarding concussion knowledge, diagnosis and management amongst emergency physicians.

**Materials and methods:**

This scoping review was conducted using the six-step process laid out by Arksey and O’Malley and included 17 papers from January 2012 to February 2023, identified by searching 5 online databases (MEDLINE, Embase, Web of Science, Scopus and Google Scholar) in February 2023 alongside a hand search of references. Search terms relevant to concussion, emergency medicine and medical education were used.

**Results:**

14 of the 17 papers originated from North America, all studies utilised either an online survey or chart review methodology. 3 papers included an educational intervention. 12 studies looked at all grades of EMPs. 14 of the studies highlighted knowledge gaps amongst EMPs, the 3 that did not specifically mention this were the 3 interventional studies.

**Conclusion:**

EMPs have large knowledge gaps regarding concussion and limited adherence to current guidelines. Efforts should be made at improving these results amongst EMPs. Further research is needed to find the most beneficial and cost-effective approach to improving concussion knowledge of concussion diagnosis and management in EDs, particularly within the UK.

**Supplementary Information:**

The online version contains supplementary material available at 10.1186/s13102-025-01091-9.

## Introduction

The definition of concussion has undergone several revisions, the most recent of which was at the 6th International Conference on Concussion in Sports Group (CISG) held in October 2022, with publication in June 2023 and is currently defined as a “traumatic brain injury caused by a direct blow to the head, neck or body resulting in an impulsive force being transmitted to the brain” [1]. Concussions are classified as a mild traumatic brain injury (mTBI) and form a subset of injuries within this category [1–3].

Concussions and head injuries are common, with previous studies in the United Kingdom (UK) estimating that somewhere between 1.2% and 6.6% of all emergency department presentations are related to head injury (through a multitude of mechanisms, not limited to sports) and they are a growing public health concern [4–6]. It has been reported that the rates of concussion are rising year on year, however this may be attributable to increased awareness of the condition by clinicians and the public, particularly through educational efforts [7–9]. It is therefore vital that emergency medicine physicians (EMPs) have an in-depth knowledge of concussion and its management. There is currently little literature on UK emergency physicians’ understanding and management of concussion, although literature has shown there to be knowledge gaps in concussion diagnosis and management within emergency departments internationally [10].

Many patients will recover symptomatically from a concussion within 10 days; however, reports suggest that 10–15% will develop persistent symptoms beyond 4 weeks [10, 11]. Persisting concussion symptoms can include: headaches, fatigue, changes in mental health (e.g. anxiousness) and reduced ability to concentrate [8]. Other authors have reported an even higher incidence of persistent symptoms. Indeed, Ingebrigtsen et al. reported that over half of adults presenting to ED with concussion will experience concussion-related symptoms at three months post injury, with as many as 30% experiencing symptoms at 1 year [12]. A study of 202 patients with concussion by Ponsford et al. suggests that early intervention (defined as neurophysiological assessment and advice within one week) can help reduce symptoms experienced at 3 months [13].

In July 2021, The Department for Digital, Culture, Media and Sport (DCMS) released a report on concussion in the community titled, “Concussion in Sport”. The report called upon the government to act on concussions and its potential “long-term neurological issues” [14]. In response to this evidence the UK Government released a report in December 2021 titled, “Government response to DCMS Select Committee report on concussion in sport”. The aim of this report is to reduce the risk of head injuries and concussion in sport. Within the Government report it is also stated that NHS England in collaboration with Faculty of Sports and Exercise Medicine prepare a learning module that will be mandatory for all GPs and A&E physicians and will need to be repeated every 2 years, to date the authors could find no evidence of this being in place [15].

Additionally, first published in June 2015 and updated in April 2023 in response to the DCMS report, the Sports and Recreation Alliance published guidelines on the management of concussion in non-elite (grassroots) sport and these have been endorsed by the Royal College of Emergency Medicine. Within these guidelines it is suggested that all children receiving a sports related head injury are assessed by an appropriate healthcare professional within 24 h [16]. Thus, patients with suspected concussion may present in varying ways and to varying physicians, notably emergency medicine, general practice, neurology, sports medicine and paediatrics [6].

Many concussions are managed by non-specialists, including EMPs and community health practitioners. It is therefore essential that those managing patients with concussion are aware of current guidelines on diagnosis and management and can offer a safe standard of care. There is also the potential of improving the recovery time and reducing persistent symptoms of concussions, although the transition from concussion to post-concussion syndrome is poorly understood [1, 14, 15, 17–19].

There have been other public health campaigns in other countries, with the aim of improving not only public awareness but physician awareness of concussion. To date the literature is limited on how effective these campaigns have been for emergency physicians [20, 21].

A recent scoping review on undergraduate medical education by Gardner and Heron concluded that medical students were under prepared for managing patients with a concussion and suggested an insufficient amount of teaching time and exposure to concussion cases during medical school. The authors concluded that this could be improved not only by increased teaching time but with improved teaching methods [6]. A review of EMP training on mTBI by Patrick et al. suggested that EMP mTBI training should fill in the gaps from medical school, but this requires physician engagement. They only found 5 articles relating to the subject (none of which were of high-quality) and that EMPs had training from resources such as training toolkits, conference presentations, journal articles or fellowship training. They stated that there is a current ‘evidence-to-practice gap’ and optimised knowledge translation required optimisation [10].

Given the reasons detailed above, this scoping review therefore aims to assess ‘what is the current level of knowledge of concussion amongst emergency medicine physicians and what knowledge gaps exist’?

## Methods

The scoping review has been based on the principles laid out by Levac et al. and Arskey and O’Malley and was done in accordance with PRSIMA (Preferred Reporting Items for Systematic Reviews and Meta-Analysis) [22, 23]. The aim of the study was to thematically present all available data relating to concussion knowledge amongst emergency medicine physicians (EMPs) and answer the question, ‘what is the current level of knowledge of concussion amongst emergency medicine physicians and what knowledge gaps exist’?

The research project was proposed by NH, who acted in a supervisory role to AG. AG carried out searches using 5 databases; MEDLINE, Embase, Scopus, Web of Science and Google Scholar. Searches were carried out in February 2023. The following searches terms were used; concussion, post-concussion syndrome, minor head injury, sports related concussion, mild traumatic brain injury, which were combined with, medical education, post graduate education, which were combined with emergency medicine, emergency physician, and emergency residency. A full copy of search terms used for each database can be found in Appendix 1. Only full text papers and those published in English were included, there were no geographical exclusions. Papers could be included if EMPs made up part of the study populations but were excluded if it was not clear what data related to EMPs. Only papers post January 2012 were included as, in 2012, with the 4th International Conference on Concussion in Sport, the 4th Consensus Statement on Concussion in Sport was subsequently published, with the first advice on return to learn (RTL), it was also the most recent CISG statement prior to the previous NICE Guidelines on head injury published in 2014, CG 176, which at the time of starting the review, were the current NICE guidelines, which have been subsequently updated in May 2023, NG 232 [11, 24]. It was therefore felt that papers post this date would most accurately reflect current day practice within emergency medicine. For those unfamiliar with RTL guidelines, RTL describes the transition back to learning following a concussion, and is of particular importance to children, adolescents, young adults, those with high acute symptom severity and those with a known learning disability. EMPs should recommend a period of 24–48 h of relative rest rather than complete rest; to try and minimise social and academic disruptions post injury, with an incremental increase in cognitive activities (total of 4 steps) and should complete the RTL prior to returning to sport [1].

.

This gave a total of 382 papers which underwent title and abstract review by AG and NH, relevant papers also had their references reviewed in the same process. This led to a total of 1014 papers screened. 23 of which underwent a full review, 6 of these were excluded; 3 were not relevant to EM, 2 we were unable to acquire a full text and 1 as it did not have specific results for EM (papers were included where EMPs were part of the study population only if it was clear which data related to the EMP group). A PRISMA diagram of the above is available in Appendix 2. This gave a total of 17 papers to be included in our review. There was clear agreement between the authors on what papers were included and excluded and each paper was assessed using checklists from the Joanna Briggs Institute (JBI) [25].

A full list of papers included in the review can be found in Appendix 3.

Once the 17 papers were identified the data charting process began. Papers were screened for common themes and after several drafts the following questions were considered (the reason for each specific question is included in brackets);

1. When and where was the research carried out? (To aid study identification and assess applicability to UK EMPs and identify geographically research active areas)
2. What was the study population? (to assess applicability to UK EMPs)
3. What methods were used? (To aid appraisal of each study and to determine what methods are currently used in the literature)
4. Was there an educational intervention and if so what was it? (To identify if any educational interventions had benefit)
5. What were the results? (To document the reported results)
6. What were the findings related to the review? (To document findings within each study)
7. What were the findings related to medical education? (To help highlight what educational findings may be of benefit to EMPs)
8. What were the limitations? (To highlight the limitations of each individual study and as such how the results should be interpreted)

## Results

Our data extraction table can be found in Appendix 4, detailing the results for each study individually. A total of 17 papers were included in this scoping review [8, 9, 20, 26–39], all were original research papers published in English between December 2012 and August 2021 involving EMPs. The majority of the papers were published from North America (USA or Canada), with 8 from the USA [8, 20, 26, 32–34, 37, 39], and 6 from Canada [27–31, 35]. The remaining 3 were from Australia [9], Singapore [36] and the UK [38] (1 from each country). All grades of EMPs were studied in 13 of the papers [8, 9, 20, 26–29, 31, 35–39], 1 studied ED Directors only [34], 1 studied attendings only [30], 1 studied residents [32] only and the remaining paper studied interns only [33].

EMPs were the sole study population for 12 [8, 9, 20, 29, 30, 33–39] of the studies and made-up part of the study group in the remaining 5 [26–28, 31, 32]. A total of 9 of the studies were single centre [8, 9, 20, 29, 32, 33, 36, 37, 39] and 5 were from a population of paediatric EMPs only [8, 26, 29, 30, 33]. From 7 of the papers, it was unclear how many EMPs were involved [8, 9, 20, 29, 35, 37, 39]. From the remaining 10 papers, a total of 722 EMPs were studied [26–28, 30–34, 36, 38] ranging from 14 (Harwayne-Gidansky et al., 2017) [33] to 158 (Carson et al., 2016) [31], with 4 having over 100 EMPs [27, 30, 31, 38]. All papers used 1 of 2 methods, either an online survey/questionnaire, 9 [26–28, 30–32, 34, 36, 38], or chart review, 8 [8, 9, 20, 29, 33, 35, 37, 39]. Only 3 of the papers had an educational intervention [32, 33, 39].

The average number of respondents for the online questionnaires was 78.67 EMPs. For the questionnaires, the response rate varied from 4 − 80%. It should be noted that Zemek et al., 2014, sent their questionnaire to a large number of potential respondents, some of whom may have been ineligible [27]. Excluding this paper there was an average response rate of 48.4% [26, 28, 29, 30, 31, 32, 35, 38]. Current guideline (at time of each study) adherence was assessed by 7 papers (1 of which was self-reported, the rest were assessed via multiple choice questions or free text responses) [27–30, 32, 35], 4 papers enquired specifically about use of assessment tools [27, 28, 30, 31]. Barriers relating to concussion were assessed in 3 papers, responses across the papers related to a lack of specialists to refer to, difficulties regarding guideline implementation, lack of training and a lack of time to educate families [26, 27, 30].

Within the 8 papers using chart reviews a total of 4,787 charts were reviewed ranging from 98 to 1855, with an average of 598 and a median of 496 charts per paper [8, 9, 20, 29, 33, 35, 37, 39]. Half were retrospective studies [8, 9, 20, 39], with the other half being prospective [25, 29, 33, 37]. There was a wide range of inclusion percentage of charts screened from 0.13% to 100%. There were varying screening methods, papers such as Koval et al., 2020 [37], and Upchurch et al., 2015 [8], screened all ED attendances whereas Yengo-Kahn et al., 2021 [39], screened only those diagnosed with concussion diagnosis on discharge. The most common reason for exclusion was lack of consent, followed by missing charts or charts with missing documentation.

From the 8 chart review papers (8, 9, 20, 29, 33, 35, 37,39), 5 looked at discharge advice and all reported this as inadequate, Koval et al. reporting 41.5% received discharge education and Yengo-Kahn et al. reporting only 3% received RTL advice prior to intervention [8, 9, 20, 37, 39]. Guideline adherence was addressed by 3 of the papers and all found inadequate adherence [9, 29, 37], 2 of the papers assessed accurate diagnosis of concussion with Boutis et al. reporting only 45% of those meeting criteria for diagnosis, receiving a diagnosis of concussion [29, 35].

Of the 17 studies included, 14 referred to either knowledge gaps, poor compliance or need for further training amongst EMPs [8, 9, 20, 26–31, 34–38]. The 3 studies which did not refer to one of the above terms were Haider et al., 2017, Harwayne-Gidansky et al., 2017 and Yengo-Kahn et al., 2021 [32, 33, 39]. These were the only 3 studies which included an educational intervention, all of which showed an improvement in performance following an educational intervention. However, EMPs were not included in the intervention group within Haider et al.’s, 2017, study, the study was still included as the data was felt to be of benefit to assessing current knowledge amongst EMPs [32].

Haider et al., 2017, studied knowledge of concussion amongst residents and students in different specialties and if a passive or active educational approach was of more benefit to learning. EMPs formed part of the control group (passive) and had the highest initial test scores (compared to family medicine, sports medicine, paediatric medicine, and medical students), the EMPs showed no improvement with passive learning strategies. Passive learning was described as "usual training", with a reading list provided [32].

Harwayne-Gidansky et al., 2017, was a randomised control trial where 20 paediatric EM interns were randomised to an immersive simulation on head injury decision rules (intervention) vs. an immersive simulation on head trauma with intracranial hypertension (control). When studied after the education sessions, residents in the intervention group were more likely to adhere to guidelines (64% vs. 43%) and performed to a level similar to senior residents [33].

Yengo-Kahn et al., 2021, was an observation pre/post intervention study in a paediatric ED. The intervention was educational and involved slide based lectures to both medical and nursing staff alongside updating the ED’s discharge paperwork. Improvements were seen post intervention, suggesting education can improve physician performance, the authors state it was unclear if the improvement was due to education or discharge paperwork [39].

## Discussion

Our study shows that there are large knowledge gaps relating to concussion knowledge amongst EMPs. These findings are not new, in 2011 Giebel et al. reported almost 75% of EMPs were not using any nationally recognised guidelines in their evaluation [40]. In 2005, after analysing 25,000 attendances in the USA from 1998 to 2000, Bazarian et al. concluded that there were substantial variations in practice in important areas of care, suggesting management of mTBI could be improved [40, 41]. The evidence included in our review is not of high quality. It is unclear why the majority of the literature comes from North America, it may be attributable to more successful public health campaigns such as HeadsUp and Think First [https://www.thinkfirst.org/our-history https://www.cdc.gov/headsup/about/index.htmlhttps://www.headsupcan.ca/about-us]. With only 1 study performed in the UK included, we need further information on how UK EMPs are performing on concussion diagnosis, management and what barriers exist to further educational efforts [38].

In 2017 Patrick et al., 2017 published a systematic review of EMP training on mild traumatic brain injury and only found 5 papers relating to their study (none of high quality), again highlighting a lack of available evidence and attention on the topic. They concluded that the current ‘evidence-to-practice gap’ put patients at risk of suboptimal care and that current education strategies for EMPs needed to be optimised [10].

It remains unclear is how this knowledge gap should be addressed, with only 2 of the studies included using an educational intervention involving EMPs. From Harwayne-Gidansky et al., whilst suggestive of potential improvement with simulation, the generalisability to EMPs is limited as they only studied interns and from Yengo-Kahn et al. we are unable to determine which intervention led to the reported increase in knowledge [33, 39]. Whilst EMPs were not in the interventional group in Haider et al.’s study, it does offer some insight that passive measures such as usual training and reading lists may not be of benefit and focus should be elsewhere [32]. As such, we are unable to recommend any singular educational intervention and suggest that further research is urgently needed in this area.

Increasing teaching not only within EM training programmes but within medical school may be required. This is a position supported in a recent scoping review on the topic by Gardner et al., 2022, who suggested that medical students’ knowledge can be improved with increased exposure to concussion cases and teaching. It should be noted that within this study emergency medicine clinical rotations are highlighted as important for student learning regarding concussion, strengthening the call for increased EMP knowledge to ensure adequate education for medical students and subsequently have less gaps to fill for EMPs of the future [6, 10].

ED attendances may continue to increase alongside raised public awareness, through drives such as concussion substitutes in professional sports, notably the addition to the Premier League in February 2021 and the use of the ‘Head Injury Assessment’ (HIA) in professional rugby since 2014 [42, 43], with public attention to sports stars being specifically mentioned in the DCMS report in 2021 [14]. This year, April 2023, the UK Government and the Sports and Recreation Alliance produced guidelines endorsed by RCEM, titled ‘If In Doubt, Sit Them Out’, on how to recognise concussion for non-elite sport. Within the document it states that anyone with a suspected concussion should be assessed by an appropriate professional within 24 h of the incident [15, 16]. The knock-on effect this will have on ED attendances is yet to be seen but may well lead to a further rise in head injury and concussion attendances due to an increased awareness.

Typically, EDs are focussed on providing emergency care to patients. Regarding head injuries, this is ensuring that patients do not have any need for neurosurgical intervention [24]. We argue that EMPs are uniquely positioned to assess and manage patients presenting with concussion or an injury that may lead to concussion and as such should have an increased awareness and knowledge on how to ensure ongoing management to prevent future morbidity. At present there are no guidelines from the RCEM requiring trainees to have specific training on concussion [44]. Given the high frequency of presentations related to head injury to UK emergency departments, we believe that this should be addressed to ensure that patients presenting to an ED receive consistent and up-to-date treatment and advice.

### Limitations

An online questionnaire method was used by 9 of the papers, and as such are subject to self-selection and recall bias [26–28, 30–32, 34, 36, 38]. It is also likely that participants completing the questionnaire would have had access to the internet whilst completing the studies and cannot be sure that they were unable to look up answers whilst completing questionnaires and potentially leading to an underestimation of the problem. There are varying methods of assessment of knowledge within the literature, with no gold standard of assessment available. Additionally, there is no clearly defined acceptable knowledge for concussion for an EMP.

It is possible that some literature may have been missed, notably grey literature, to limit this and endeavour to include all relevant papers meeting the inclusion criteria, a search strategy using multiple databases alongside reviewing extensive lists of references was utilised. Two researchers (AG and NH) independently reviewed each paper and reached consensus on those to be included.

## Conclusion

It is clear from the literature that EMPs are not up to date in concussion knowledge and its management or implementing current clinical guidelines. Evidence on EMP concussion knowledge is particularly lacking within the UK setting, with only 1 paper since January 2012 included in the review. We suggest that further research is needed in the area, specifically looking to address what educational tools can be utilised to improve the current situation around concussion diagnosis and management in the emergency department. Additionally, guidance from national bodies, such as RCEM, is needed to make clear what knowledge is expected from an EMP.

## Electronic supplementary material

Below is the link to the electronic supplementary material.

Supplementary Material 1

## Data Availability

All data are freely available within the publication.

## References

[1] Patricios JS, Schneider KJ, Dvorak J et al., *Consensus statement on concussion in sport: the 6th International Conference on Concussion in Sport–Amsterdam, October 2022, British Journal of Sports Medicine 2023;57:695–711* 10.1136/bjsports-2023-106898 37316210

[2] Aubry M, Cantu R, Dvorak J, Graf-Baumann T, Johnston K, Kelly J, Lovell M, McCrory P, Meeuwisse W, Schamasch P. Concussion in Sport Group. Summary and agreement statement of the First International Conference on Concussion in Sport, Vienna 2001. Recommendations for the improvement of safety and health of athletes who may suffer concussive injuries. Br J Sports Med. 2002;36(1):6–10. 10.1136/bjsm.36.1.6. PMID: 11867482; PMCID: PMC1724447. 10.1136/bjsm.36.1.6 PMC1724447 11867482

[3] McCrory P, Meeuwisse W, Dvorak J, et al. Consensus statement on concussion in sport—the 5th international conference on concussion in sport held in Berlin, October 2016. Br J Sports Med. 2017;51:838–47. 28446457 10.1136/bjsports-2017-097699

[4] Toman E, Hodgson S, Riley M, Welbury R, Di Pietro V, Belli A. Concussion in the UK: a contemporary narrative review. Trauma Surg Acute Care Open. 2022;7(1):e000929. 10.1136/tsaco-2022-000929. PMID: 36274785; PMCID: PMC9582316. 36274785 10.1136/tsaco-2022-000929 PMC9582316

[5] Marincowitz C, Lecky FE, Morris E, Allgar V, Sheldon TA. Impact of the SIGN head injury guidelines and NHS 4-hour emergency target on hospital admissions for head injury in Scotland: an interrupted times series. BMJ Open. 2018;8(12):e022279. 10.1136/bmjopen-2018-022279. PMID: 30580260; PMCID: PMC6318526. 30580260 10.1136/bmjopen-2018-022279 PMC6318526

[6] Gardner N, Heron NA, Scoping Review. Mapping the evidence for undergraduate concussion education and proposing the content for medical student concussion teaching. Int J Environ Res Public Health. 2022;19(7):4328. 10.3390/ijerph19074328. PMID: 35410008; PMCID: PMC8998836. 35410008 10.3390/ijerph19074328 PMC8998836

[7] Langer L, Levy C, Bayley M. Increasing incidence of concussion: true epidemic or better recognition?? J Head Trauma Rehabil. 2020;35(1):E60–6. 10.1097/HTR.0000000000000503. 31246881 10.1097/HTR.0000000000000503

[8] Upchurch C, Morgan CD, Umfress A, Yang G, Riederer MF. Discharge instructions for youth sports-related concussions in the emergency department, 2004 to 2012. Clin J Sport Med. 2015;25(3):297-9. 10.1097/JSM.0000000000000123. PMID: 24977953. 10.1097/JSM.0000000000000123 24977953

[9] Brown AM, Twomey DM, Wong Shee A. Evaluating mild traumatic brain injury management at a regional emergency department. Inj Prev. 2018;24(5):390–394. 10.1136/injuryprev-2018-042865. Epub 2018 Jun 4. PMID: 29866717. 10.1136/injuryprev-2018-042865 29866717

[10] Patrick SP, Gaudet LA, Krebs LD, Chambers T, Rowe BH. Emergency physician training on mild traumatic brain injury: A systematic review. AEM Educ Train. 2017;1(4):346–56. 10.1002/aet2.10053. PMID: 30051054; PMCID: PMC6001600. 30051054 10.1002/aet2.10053 PMC6001600

[11] McCrory P, Meeuwisse WH, Aubry M, Cantu RC, Dvořák J, Echemendia RJ, Engebretsen L, Johnston K, Kutcher JS, Raftery M, Sills A, Benson BW, Davis GA, Ellenbogen R, Guskiewicz KM, Herring SA, Iverson GL, Jordan BD, Kissick J, McCrea M, McIntosh AS, Maddocks D, Makdissi M, Purcell L, Putukian M, Schneider K, Tator CH, Turner M. Consensus statement on concussion in sport: the 4th International Conference on Concussion in Sport, Zurich, November 2012. J Athl Train. 2013 Jul-Aug;48(4):554– 75. 10.4085/1062-6050-48.4.05. PMID: 23855364; PMCID: PMC3715021. 10.4085/1062-6050-48.4.05 PMC3715021 23855364

[12] Ingebrigtsen T, Waterloo K, Marup-Jensen S, Attner E, Romner B. Quantification of post-concussion symptoms 3 months after minor head injury in 100 consecutive patients. J Neurol. 1998;245(9):609– 12. 10.1007/s004150050254. PMID: 9758300. 10.1007/s004150050254 9758300

[13] Ponsford J, Willmott C, Rothwell A, Cameron P, Kelly AM, Nelms R, Curran C. Impact of early intervention on outcome following mild head injury in adults. J Neurol Neurosurg Psychiatry. 2002;73(3):330–2. 10.1136/jnnp.73.3.330. PMID: 12185174; PMCID: PMC1738009. 12185174 10.1136/jnnp.73.3.330 PMC1738009

[14] House of Commons. Digital, Culture, Media and Sport Committee, Concussion in sport, Third Report of Session 2021-22, Ordered by the House of Commons to be printed 15 July 2021.

[15] Government response to DCMS Select Committee report on concussion in sport, 10. Dec 2021, https://www.gov.uk/government/publications/government-response-to-the-digital-culture-media-and-sport-select-committee-report-on-concussion-in-sport/government-response-to-dcms-select-committee-report-on-concussion-in-sport

[16] The UK Government and Sports and, Alliance R. April, If in doubt, sit them out, UK concussion guidelines for Non-Elite (Grassroots) sport, 2023; http://sramedia.s3.amazonaws.com/media/documents/9ced1e1a-5d3b-4871-9209-bff4b2575b46.pdf

[17] Mistry DA, Rainer TH. Concussion assessment in the emergency department: a preliminary study for a quality improvement project. BMJ Open Sport Exerc Med. 2018;4(1):e000445. 10.1136/bmjsem-2018-000445. PMID: 30687512; PMCID: PMC6326330. 30687512 10.1136/bmjsem-2018-000445 PMC6326330

[18] Svensson S, Vedin T, Clausen L, Larsson PA, Edelhamre M. Application of NICE or SNC guidelines May reduce the need for computerized tomographies in patients with mild traumatic brain injury: a retrospective chart review and theoretical application of five guidelines. Scand J Trauma Resusc Emerg Med. 2019;27(1):99. 10.1186/s13049-019-0673-8. PMID: 31684991; PMCID: PMC6829961. 31684991 10.1186/s13049-019-0673-8 PMC6829961

[19] Ferry B, DeCastro A. Concussion. [Updated 2022 Jan 19]. In: StatPearls [Internet]. Treasure Island(FL): StatPearls Publishing; 2022 Jan-. Available from: https://www.ncbi.nlm.nih.gov/books/NBK537017

[20] Lane AD, Berkman MR, Verbunker D, Shekell T, Bouska M, Barnett L, Keogh A, Nuno T, Stolz U, Waterbrook AL. Retrospective chart analysis of concussion discharge instructions in the emergency department. J Emerg Med. 2017;52(5):690–8. 10.1016/j.jemermed.2016.12.017. Epub 2017 Feb 13. PMID: 28202206. 28202206 10.1016/j.jemermed.2016.12.017

[21] Sarmiento K, Daugherty J, Waltzman D. Effectiveness of the CDC HEADS UP online training on healthcare providers’ mTBI knowledge and self-efficacy. J Saf Res. 2021;78:221–8. 10.1016/j.jsr.2021.04.004. Epub 2021 May 7. PMID: 34399918; PMCID: PMC8375598. 10.1016/j.jsr.2021.04.004 PMC8375598 34399918

[22] Levac D, Colquhoun H, O’Brien KK. Scoping Studes: advancing the methodology. Implement Sciene. 2010;5(69):1–9. 10.1186/1748-5908-5-69. 10.1186/1748-5908-5-69 PMC2954944 20854677

[23] Arksey H, O’Malley L. Scoping studies: towards a methodological framework. Int J Social Res Methodology: Theory Pract. 2005;8(1):19–32. 10.1080/1364557032000119616.

[24] NICE Guideline (NG232.), Head injury: assessment and early management, 18 May 2023.

[25] Joanna Briggs Institute. (2017). *Checklist for systematic reviews and research syntheses*. https://joannabriggs.org/ebp/critical_appraisal_tools

[26] Zonfrillo MR, Master CL, Grady MF, Winston FK, Callahan JM, Arbogast KB. Pediatric providers’ self-reported knowledge, practices, and attitudes about concussion. Pediatrics. 2012;130(6):1120–5. 10.1542/peds.2012-1431. Epub 2012 Nov 12. PMID: 23147981. 23147981 10.1542/peds.2012-1431

[27] Zemek R, Eady K, Moreau K, Farion KJ, Solomon B, Weiser M, Dematteo C. Knowledge of paediatric concussion among front-line primary care providers. Paediatr Child Health. 2014;19(9):475–80. 10.1093/pch/19.9.475. PMID: 25414583; PMCID: PMC4235448. 25414583 10.1093/pch/19.9.475 PMC4235448

[28] Stoller J, Carson JD, Garel A, Libfeld P, Snow CL, Law M, Frémont P. Do family physicians, emergency department physicians, and pediatricians give consistent sport-related concussion management advice? Can Fam Physician. 2014;60(6):548. PMID: 24925947; PMCID: PMC4055323. 24925947 PMC4055323

[29] Boutis K, Weerdenburg K, Koo E, Schneeweiss S, Zemek R. The diagnosis of concussion in a pediatric emergency department. J Pediatr. 2015;166(5):1214–1220.e1. 10.1016/j.jpeds.2015.02.013. PMID: 25919731. 10.1016/j.jpeds.2015.02.013 25919731

[30] Zemek R, Eady K, Moreau K, Farion KJ, Solomon B, Weiser M, Dematteo C. Canadian pediatric emergency physician knowledge of concussion diagnosis and initial management. CJEM. 2015;17(2):115– 22. 10.1017/cem.2014.38. PMID: 25927255. 10.1017/cem.2014.38 25927255

[31] Carson JD, Rendely A, Garel A, Meaney C, Stoller J, Kaicker J, Hayden L, Moineddin R, Frémont P. Are Canadian clinicians providing consistent sport-related concussion management advice? Can Fam Physician. 2016;62(6):494–500. PMID: 27303008; PMCID: PMC4907559. 27303008 PMC4907559

[32] Haider MN, Leddy JJ, Baker JG, Kiel JM, Tiso M, Ziermann KA, Willer BS. Concussion management knowledge among residents and students and how to improve it. Concussion. 2017;2(3):CNC40. 10.2217/cnc-2017-0001. PMID: 30202581; PMCID: PMC6093773. 30202581 10.2217/cnc-2017-0001 PMC6093773

[33] Harwayne-Gidansky I, Bellis JM, McLaren SH, Critelli K, Clark S, Chen Z, Gerber LM, Ching K. Mannequin-based immersive simulation improves resident Understanding of a clinical decision rule. Simul Gaming. 2017;48(5):657–69.

[34] Stern RA, Seichepine D, Tschoe C, Fritts NG, Alosco ML, Berkowitz O, Burke P, Howland J, Olshaker J, Cantu RC, Baugh CM, Holsapple JW. Concussion care practices and utilization of Evidence-Based guidelines in the evaluation and management of concussion: A survey of new England emergency departments. J Neurotrauma. 2017;34(4):861–8. 10.1089/neu.2016.4475. Epub 2016 May 19. PMID: 27112592; PMCID: PMC5314982. 27112592 10.1089/neu.2016.4475 PMC5314982

[35] Rowe BH, Eliyahu L, Lowes J, Gaudet LA, Beach J, Mrazik M, Cummings G, Voaklander D. Concussion diagnoses among adults presenting to three Canadian emergency departments: missed opportunities. Am J Emerg Med. 2018;36(12):2144–51. 10.1016/j.ajem.2018.03.040. Epub 2018 Mar 20. PMID: 29636295. 29636295 10.1016/j.ajem.2018.03.040

[36] Sirisena D, Walter J, Ong JH, Probert J. Pilot single-centre cross-sectional study to determine emergency physicians’ knowledge and management of sports concussion: an experience from Singapore. Singap Med J. 2018;59(6):322–6. 10.11622/smedj.2017104. Epub 2017 Nov 13. PMID: 29167908; PMCID: PMC6024222. 10.11622/smedj.2017104 PMC6024222 29167908

[37] Koval RR, Zalesky CC, Moran TP, Moore JC, Ratcliff JJ, Wu DT, Wright DW. Concussion Care in the Emergency Department: A Prospective Observational Brief Report. Ann Emerg Med. 2020;75(4):483–490. 10.1016/j.annemergmed.2019.08.4192019.08.419. Epub 2019 Nov 1. Erratum in: Ann Emerg Med. 2020;76(3):377. PMID: 31685254. 10.1016/j.annemergmed.2019.08.419 31685254

[38] Rashid H, Mishra S, Dobbin N. Management of sport-related concussion in emergency departments in England: a multi-center study. Brain Inj. 2021;35(9):1035–42. 10.1080/02699052.2021.1945146. Epub 2021 Jul 21. PMID: 34288793. 34288793 10.1080/02699052.2021.1945146

[39] Yengo-Kahn AM, Hibshman N, Bezzerides M, Feldman MJ, Vukovic AA, Mummareddy N, Zhao S, Penrod CH, Bonfield CM, Vance EH. Improving discharge instructions following a concussion diagnosis in the pediatric emergency department: A Pre-post intervention study. Pediatr Qual Saf. 2021;6(5):e456. 10.1097/pq9.0000000000000456. PMID: 34476308; PMCID: PMC8389964. 34476308 10.1097/pq9.0000000000000456 PMC8389964

[40] Giebel S, Kothari R, Koestner A, Mohney G, Baker R. Factors influencing emergency medicine physicians’ management of sports-related concussions: a community-wide study. J Emerg Med. 2011;41(6):649–54. 10.1016/j.jemermed.2011.03.021. Epub 2011 May 7. PMID: 21550754. 21550754 10.1016/j.jemermed.2011.03.021

[41] Bazarian JJ, McClung J, Cheng YT, Flesher W, Schneider SM. Emergency department management of mild traumatic brain injury in the USA. Emerg Med J. 2005;22(7):473–7. 10.1136/emj.2004.019273. PMID: 15983080; PMCID:PMC1726852. 15983080 10.1136/emj.2004.019273 PMC1726852

[42] Tarzi G, Tarzi C, Mirsu D, Patel J, Dadashi E, El-Sabbagh J, Gerhart A, Cusimano MD. Effect of a new concussion substitute rule on medical assessment of head collision events in premier league football. Inj Prev. 2022;28(6):521–5. 10.1136/ip-2022-044580. Epub 2022 Jul 5. PMID: 35790348; PMCID: PMC9726948. 35790348 10.1136/ip-2022-044580 PMC9726948

[43] Falvey É, Tucker R, Fuller G, Raftery M. Head injury assessment in rugby union: clinical judgement guidelines. BMJ Open Sport Exerc Med. 2021;7(2):e000986. 10.1136/bmjsem-2020-000986. PMID: 33981448; PMCID: PMC8061850. 33981448 10.1136/bmjsem-2020-000986 PMC8061850

[44] Curriculum. 2021, The Royal College of Emergency Medicine, August 2021. https://acem.org.au/getmedia/9af41df8-677f-44ed-b245-440164155f56/FACEM-Curriculum-2021

