# Supplementary material for: Heads up for concussion, what do emergency physicians know? A scoping review
Source: BMC Sports Sci Med Rehabil. 2025 Mar 27;17:61. doi: 10.1186/s13102-025-01091-9 (PMC11948858; doi:10.1186/s13102-025-01091-9)
Supplement: Supplementary file 1 — Supplementary Material 1 [file 13102_2025_1091_MOESM1_ESM.docx]

**Supplementary Data**

**Appendix 1: List of search terms used in databases**

**
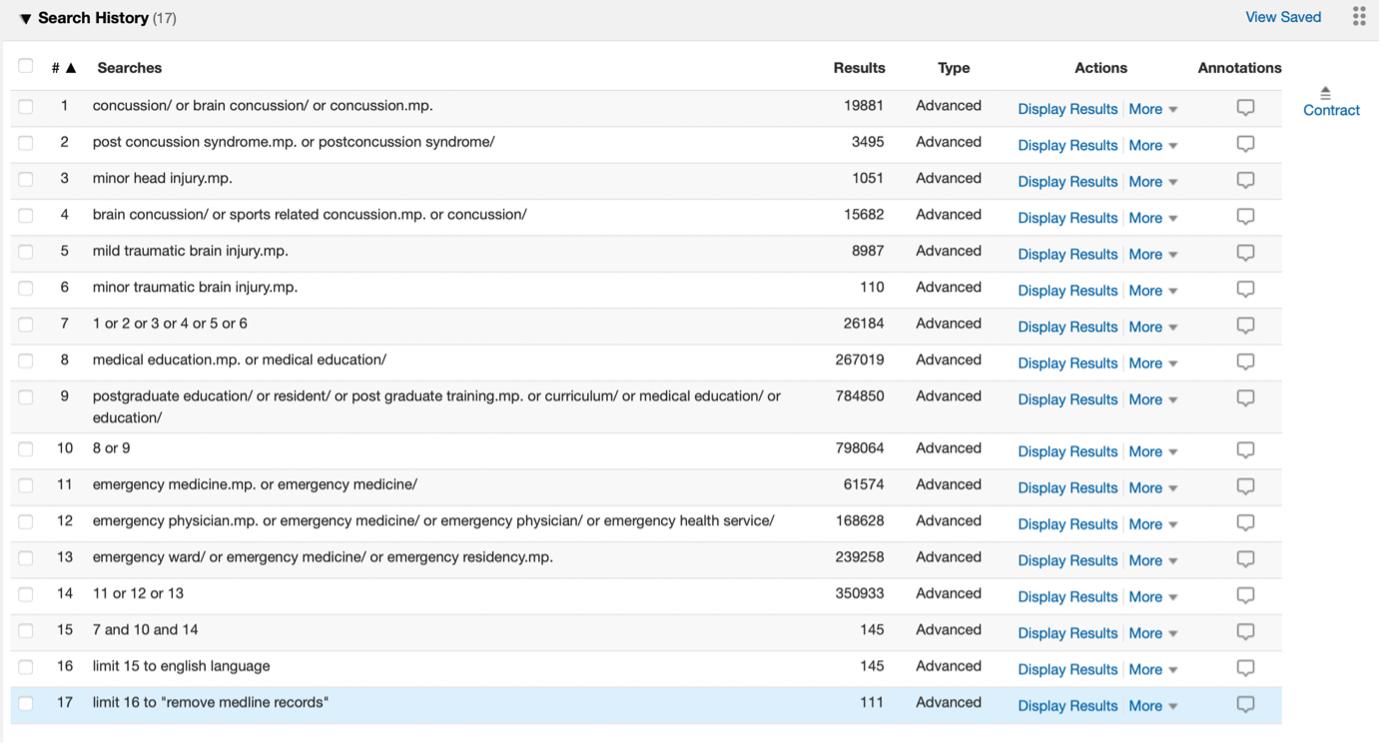
**

**Appendix 2: PRISMA Diagram for scoping review**

**Identification of studies via databases and registers**

Hand screening of references

(n= 632)

Records identified from*: 382

Databases (n = 5 )

Registers (n =0 )

**Identification**

Records excluded inc duplicates**

(n = 991)

Records screened

(n = 1014)

**Screening**

Reports excluded: 6

- Reason 1 (n = 2) not relevant to EM practice
- Reason 2 (n = 1) not related to EM physician practice
- Reason 3 (n = 1) not clear which data relates to EM
- Reason 4 (n = 2) unable to get full text

Reports assessed for eligibility

(n = 23)

Studies excluded from review

(n = 370 + 300 + 1 + 210 + 2 + 111 + 1 = 995)

Studies included in review

(n = 17)

**Included**

**Excluded**

*From:*  Page MJ, McKenzie JE, Bossuyt PM, Boutron I, Hoffmann TC, Mulrow CD, et al. The PRISMA 2020 statement: an updated guideline for reporting systematic reviews. BMJ 2021;372:n71. Doi: 10.1136/bmj.n71

**Appendix 3: References for papers within scoping review**

1. Zonfrillo MR, Master CL, Grady MF, Winston FK, Callahan JM, Arbogast KB. Pediatric providers’ self-reported knowledge, practices, and attitudes about concussion. Pediatrics. 2012 Dec;130(6):1120-5. Doi: 10.1542/peds.2012-1431. Epub 2012 Nov 12. PMID: 23147981.
2. Zemek R, Eady K, Moreau K, Farion KJ, Solomon B, Weiser M, Dematteo C. Knowledge of paediatric concussion among front-line primary care providers. Paediatr Child Health. 2014 Nov;19(9):475-80. Doi: 10.1093/pch/19.9.475. PMID: 25414583; PMCID: PMC4235448.
3. Stoller J, Carson JD, Garel A, Libfeld P, Snow CL, Law M, Frémont P. Do family physicians, emergency department physicians, and pediatricians give consistent sport-related concussion management advice? Can Fam Physician. 2014 Jun;60(6):548, 550-2. PMID: 24925947; PMCID: PMC4055323.
4. Boutis K, Weerdenburg K, Koo E, Schneeweiss S, Zemek R. The diagnosis of concussion in a pediatric emergency department. J Pediatr. 2015 May;166(5):1214-1220.e1. doi: 10.1016/j.jpeds.2015.02.013. PMID: 25919731.
5. Upchurch C, Morgan CD, Umfress A, Yang G, Riederer MF. Discharge instructions for youth sports-related concussions in the emergency department, 2004 to 2012. Clin J Sport Med. 2015 May;25(3):297-9. Doi: 10.1097/JSM.0000000000000123. PMID: 24977953.
6. Zemek R, Eady K, Moreau K, Farion KJ, Solomon B, Weiser M, Dematteo C. Canadian pediatric emergency physician knowledge of concussion diagnosis and initial management. CJEM. 2015 Mar;17(2):115-22. Doi: 10.1017/cem.2014.38. PMID: 25927255.
7. Carson JD, Rendely A, Garel A, Meaney C, Stoller J, Kaicker J, Hayden L, Moineddin R, Frémont P. Are Canadian clinicians providing consistent sport-related concussion management advice? Can Fam Physician. 2016 Jun;62(6):494-500. PMID: 27303008; PMCID: PMC4907559.
8. Haider MN, Leddy JJ, Baker JG, Kiel JM, Tiso M, Ziermann KA, Willer BS. Concussion management knowledge among residents and students and how to improve it. Concussion. 2017 Aug 3;2(3):CNC40. Doi: 10.2217/cnc-2017-0001. PMID: 30202581; PMCID: PMC6093773.
9. Harwayne-Gidansky I, Bellis JM, McLaren SH, Critelli K, Clark S, Chen Z, Gerber LM, Ching K. Mannequin-based immersive simulation improves resident understanding of a clinical decision rule. Simulation & Gaming. 2017 Oct;48(5):657-69.
10. Lane AD, Berkman MR, Verbunker D, Shekell T, Bouska M, Barnett L, Keogh A, Nuno T, Stolz U, Waterbrook AL. Retrospective Chart Analysis of Concussion Discharge Instructions in the Emergency Department. J Emerg Med. 2017 May;52(5):690-698. Doi: 10.1016/j.jemermed.2016.12.017. Epub 2017 Feb 13. PMID: 28202206.
11. Stern RA, Seichepine D, Tschoe C, Fritts NG, Alosco ML, Berkowitz O, Burke P, Howland J, Olshaker J, Cantu RC, Baugh CM, Holsapple JW. Concussion Care Practices and Utilization of Evidence-Based Guidelines in the Evaluation and Management of Concussion: A Survey of New England Emergency Departments. J Neurotrauma. 2017 Feb 15;34(4):861-868. Doi: 10.1089/neu.2016.4475. Epub 2016 May 19. PMID: 27112592; PMCID: PMC5314982.
12. Brown AM, Twomey DM, Wong Shee A. Evaluating mild traumatic brain injury management at a regional emergency department. Inj Prev. 2018 Oct;24(5):390-394. Doi: 10.1136/injuryprev-2018-042865. Epub 2018 Jun 4. PMID: 29866717.
13. Rowe BH, Eliyahu L, Lowes J, Gaudet LA, Beach J, Mrazik M, Cummings G, Voaklander D. Concussion diagnoses among adults presenting to three Canadian emergency departments: Missed opportunities. Am J Emerg Med. 2018 Dec;36(12):2144-2151. Doi: 10.1016/j.ajem.2018.03.040. Epub 2018 Mar 20. PMID: 29636295.
14. Sirisena D, Walter J, Ong JH, Probert J. Pilot single-centre cross-sectional study to determine emergency physicians’ knowledge and management of sports concussion: an experience from Singapore. Singapore Med J. 2018 Jun;59(6):322-326. Doi: 10.11622/smedj.2017104. Epub 2017 Nov 13. PMID: 29167908; PMCID: PMC6024222.
15. Koval RR, Zalesky CC, Moran TP, Moore JC, Ratcliff JJ, Wu DT, Wright DW. Concussion Care in the Emergency Department: A Prospective Observational Brief Report. Ann Emerg Med. 2020 Apr;75(4):483-490. Doi: 10.1016/j.annemergmed.2019.08.419. Epub 2019 Nov 1. Erratum in: Ann Emerg Med. 2020 Sep;76(3):377. PMID: 31685254.
16. Rashid H, Mishra S, Dobbin N. Management of sport-related concussion in emergency departments in England: a multi-center study. Brain Inj. 2021 Jul 29;35(9):1035-1042. Doi: 10.1080/02699052.2021.1945146. Epub 2021 Jul 21. PMID: 34288793.
17. Yengo-Kahn AM, Hibshman N, Bezzerides M, Feldman MJ, Vukovic AA, Mummareddy N, Zhao S, Penrod CH, Bonfield CM, Vance EH. Improving Discharge Instructions Following a Concussion Diagnosis in the Pediatric Emergency Department: A Pre-post Intervention Study. Pediatr Qual Saf. 2021 Aug 26;6(5):e456. Doi: 10.1097/pq9.0000000000000456. PMID: 34476308; PMCID: PMC8389964

Appendix 4: Data Extraction Table

| Author (reference) | Year and Journal | Country | Population and ED setting | Methods | Intervention | Results | Key findings related to review | Educational findings | Limitations |
| --- | --- | --- | --- | --- | --- | --- | --- | --- | --- |
| Zonfrillo MR, Master CL et al (28) | 2012, Pediatrics | USA | 57 paediatric EMPs of 145 respondents in “Philadelphia Care Network” (50 sites) | Cross-sectional electronic survey on concussion diagnosis and management | No | High rates of correct diagnosis amongst EM respondents | Majority of EM felt they had inadequate time or training in assessing concussion | Improved education methods and time spent on concussion management in the ED may be required | Only paediatric EMPs.  Comparing EM to GPs.  Potential for self-selection and recall bias.  Unvalidated questionnaire |
| Zemek R, Eady K et al (29) | 2014, Canadian Family Physician | Canada | 116 EMPs in Ontario of 753 respondents | Online survey assessing diagnosis and management of concussion | No | High rate of correct diagnosis amongst EMPs, however two thirds had inadequate knowledge discharge instructions | Knowledge gap amongst EMPs in relation to management of concussion | Education required in relation to adequate discharge advice and onward management of concussion | Open to self-selection and recall bias.  Unable to determine accurate response rate.  Not EM specific.  Unvalidated questionnaire. |
| Stoller J, Carson JD et al (30) | 2014, Canadian Family Physician | Canada | 21 EMPs from a total of 104 from 2 community teaching hospitals in Toronto | Online 19-question MCQ | No | Poor EM awareness of current guidelines relating to concussion | Large knowledge gaps identified along with inconsistent use of current recommendations | More effort needed towards knowledge translation of current recommendations | Open to self-selection and recall bias.  Not EM specific.  Small number of EMPs. |
| Boutis K, Weerdenburg K et al (31) | 2015, The Journal of Pediatrics | Canada | 495 ED visits at tertiary care paediatric ED | Prospective study comparing rate of concussion diagnosis on ED record vs current guidelines | No | Only 40.4% correct diagnosis rate (200 out of 443) | Underdiagnosis of concussion suggests lack of knowledge from paediatric EMPs | More education need with regard to current criteria needed for diagnosis | Single centre, tertiary care ED.  Paediatric population only.  Patients reviewed prior to EMP by research assistants which may have led to Hawthorne effect.  No patients included overnight (convenience sample). |
| Upchurch, Cameron BS et al (13) | 2015, Clinical Journal of Sports Medicine | USA | 497 chart reviews at single tertiary care paediatric ED | Retrospective chart review of patients diagnosed with SRC over 8-year period | No | Only 66% received adequate discharge advice despite the CDC’s “Heads Up Campaign” | Significant proportion of patients not having adequate ongoing management suggests need for EMP education | Lack of awareness amongst EMPs regarding adequate discharge planning and further educational efforts should be made to this regard | Only 497 SRCs in 392,908 attendances, likely missed many cases.  Single tertiary care centre.  Limited to note review. Retrospective data. |
| Zemek R, Eady K et al (32) | 2015, Canadian Journal of Emergency Medicine | Canada | 115 attending paediatric EMPs from the Pediatric Emergency Research Canada Network (15 hospitals) | 35 item online questionnaire | No | High rate of correct diagnosis but one third unable to correctly apply discharge guidelines | EMPs unable to correctly give current discharge advice to patients | Further educational efforts needed at improving EMPs awareness of discharge guidelines. | Attending physicians only.  Open to self-selection and recall bias.  Participants were members of a research network; this population may not accurately represent the EM community. |
| Carson JD, Rendely A et al (33) | 2016, Canadian Family Physician | Canada | 158 EMPs of 305 total | Online 19 MCQ questionnaire | No | EMPs worse at managing concussion than sports medicine physicians, EMPs preferred learning from journals and conferences. | Large knowledge gaps and inconsistent adherence to guidelines amongst EMPs | Need for further EMP education and research into which knowledge translational strategies are most beneficial.  EMPs prefer learning from journals/conferences over public health website aimed at learning. | Poor response rate from EMPs.  Open to self-selection and recall bias.  Very limited learning preference options: journal, conference or “ThinkFirst Canada” website. |
| Haider M, Leddy LJ et al (34) | 2017, Concussion | USA | 14 EM residents as part of control group along with paediatrics and GP vs active learning group of sports medicine and medical students. | Pre and post-concussion knowledge questionnaire | EM were on control group which was described as usual training and given a short list of reading material, intervention group had “active” educational intervention which was described as participation in 2 half day concussion clinic sessions a week for 4 weeks | EM had highest baseline scores and highest exposure to concussion.  EM had no improvement in scores despite passive measures.  Active learners had significantly improved results compared to passive learners. | Passive learning methods had no benefit for EMPs.  Active learning would appear to improve knowledge and management of concussion vs passive learning, although this study cannot say this is true for EMPs. | Educational strategies for EM should consider incorporating active learning elements | Single centre. Small number of EMPs.  No EM representation in active learner group so results may not be applicable to EMPs.  No control and active learners of the same speciality.  Applicability of sending EM trainees to outpatient concussion clinics?  No randomisation in groups.  Active group included Sports Medicine Residents, who by nature of their specialty may have been keener to learn about concussion.  49% loss to follow up. |
| Harwayne-Gidansky I, Bellis JM et al (35) | 2017, Simulation and Gaming | USA | 20 paediatric EM interns | Single centre, simulation based, blinded randomised control trial. hypertension. Subsequently collected data from EMPs future notes on patients with minor head injury. | Participants randomised to either clinical teaching with simulation on minor head trauma and PECARN* rule vs clinical teaching with simulation on intracranial | Improved adherence to guidelines in intervention group, they performed to a level several years above their current grade. | Simulation scenarios on decision making, diagnosis and management of head injuries in the ED can be improved with simulation and potentially advance EMPs decision making to that of a higher grade. | EM curriculums should aim to have simulation training on head injuries presenting to ED and their onward disposal. | Single tertiary care centre.  Small numbers.  Unblinded  Required EMPs to fill in extra sheet in notes for study purposes for each patient presenting with head trauma, it is unclear what percentage this was done for as only 144 were completed over a one-year period.  Selection bias may be present when extra form is needed. |
| Lane A, Berkman MR et al (23) | 2017, Journal of Emergency Meidicne | USA | 1855 chart reviews in a level 1 trauma centre in USA | 1 year chart review of discharge instructions for patients discharged with a closed head injury | No | Only 71% received discharge advice, those with SRCs were more likely to receive advice. RTP advice only given to 29% of SRCs | Significant amount of patients discharged with no advice. Vast majority of patients not receiving adequate RTP advice | Further education is needed aimed at EMP discharge of patients with concussion. | Single centre tertiary care centre.  Study was a quality improvement project.  Limited to note review.  Retrospective data |
| Stern RA, Seichepine at al (36) | 2017, Journal of Neurotrauma | USA | 67 ED directors | Online 32 question survey | No | 35% of EDs did not use guidelines in management of concussion, of the 65%, 57% reported inconsistent use. Clinicians mostly used clinical examination over decision rules regarding decision to request neuroimaging. | Poor utilisation of guidelines and clinical decision rules may represent a lack of knowledge | There is a need to improve knowledge translation from clinical guidelines to practice. | Open to self-selection and recall bias.  Under-representation of rural hospitals.  Only senior EMPs surveyed and only one from each hospital, is this representative of the average EMP? |
| Brown AM, Twomey DM et al (14) | 2018, Injury Prevention | Australia | 540 ED records of 16+ year olds presenting with mTBI | Retrospective chart review audit | No | 74.1% had documented safe discharge, 33% received discharge education. Only 65.3% of those meeting criteria for CT had one performed. | Lack of adherence to clinical guidelines may represent knowledge gaps within this population. | Further efforts are needed at knowledge translation from guidelines to clinical practice. | Retrospective.  Single centre.  Review of handwritten notes.  States that there was potential for missing charts. |
| Rowe BH, Eliyahu L et al (37) | 2018, American Journal of Emergency Medicine | Canada | 250 adult ED patients at 3 different sites. | Prospective observational cohort study, measuring proportion of patients that met WHO criteria for concussion who were correctly diagnosed by the treating EMP. | No | 16% who met criteria were misdiagnosed in the ED.  Those who were correctly diagnosed spent less time in ED | Apparent knowledge gap in EMPs diagnosing concussion, seemingly different presentations e.g. RTC related head injury were more at risk of misdiagnosis. | Further efforts are needed at educating EMPs and range of presenting mechanism of injuries for concussion, this interestingly may reduce ED LOS for these patients. | Not 24-hour research cover so may have missed patients, convenience sample.  Unable to comment on LOS overnight or at weekends.  Unaware if EDPs aware of study, if so open to Hawthorne effect.  Only included English speaking adults. |
| Sirisena D, Walter J et al (38) | 2018, Singapore Medical Journal | Singapore | 52 EDPs | 17 item online questionnaire | Nil | EDPs manage concussion on a regular basis. 75% were unaware of existing clinical guidelines. Only 50% | Limited training in concussion amongst cohort, however no significant differences between those who had and those who hadn’t had training (small numbers). | Further training in concussion is needed for EDPs and evaluation of different training methods. | Single centre.  Self-selection and recall bias. |
| Koval RR, Zaesky CC et al (39) | 2020, Annals of Emergency Medicine | USA | 98 ED records | Prospective observational study, involving ED chart review with 2 stage identification questions used at triage | Nil | <50% had adequate documentation of concussion signs and symptoms. 41.7% diagnosed received discharge instructions.  92.3% had not any formal training on concussion. | Poor documentation and lack of appropriate discharge advice would suggest inadequate knowledge | Education should highlight the importance of correct diagnosis and discharge advice | Chart review, therefore reliant on ED written notes.  Single centre.  Single unblinded reviewer.  Unvalidated screening questions.  May have missed patients with concussion/mTBI. |
| Rashid H, Mishra S and Dobbin N (40) | 2021, Brain Injury | UK (England) | 102 EDPs across 15 EDs | Cross-sectional, multi- centre study using a 22-item online questionnaire | Nil | 63.6% were aware of clinical guidelines regarding SRC. 57% were aware of graduated return-to-sport, 42% provided written discharge instructions.  6% had previous training as part of their EM training, with 13.6% having training outside ED, with 86.4% with no training. 95% felt should be part of training. | Lack of knowledge of guidelines would suggest a knowledge gap amongst respondents. Respondents were overwhelmingly in favour of having formalised concussion training as part of EM training. | More efforts should be mad in the training of EMPs regarding concussion. | Open to self-selection and recall bias.  No data amongst EDs to allow comparison between sites.  Focus solely on SRCs and not all mTBIs. |
| Yengo-Kahn AM, Hibshman N et al (41) | 2021, Paediatric Quality and Saftey | USA | 935 patients   - 375 pre - 560 post | Chart review observational pre/post intervention study | Education sessions (slide-based group lectures) and improved discharge paperwork. | Higher rates of more junior clinicians discharging post intervention.  Increased rates of RTP discharge advice 33% vs 69% and RTL from 50% to 95%.  Results worsened when there was a staff changeover | Education of EMPs lead to improved compliance with current guidelines.  Education sessions may need to be repeated, specifically at changeover times. | Education sessions may be of benefit to EDPs in improving compliance with guidelines, efforts should be made regularly but particular importance should be paid to times of changeover/rotation of staff. | Single centre.  Didn’t record attendance at education sessions.  Unclear if improvement secondary to education or improved discharge advice.  Unclear what educational methods would prove most beneficial.  Demographic differences between two groups.  “Excitement regarding the project” may have been confounding factor. Retrospective data. |
